# ROCK-phosphorylated vimentin modifies mutant huntingtin aggregation via sequestration of IRBIT

**DOI:** 10.1186/1750-1326-7-43

**Published:** 2012-08-28

**Authors:** Peter O Bauer, Roman Hudec, Anand Goswami, Masaru Kurosawa, Gen Matsumoto, Katsuhiko Mikoshiba, Nobuyuki Nukina

**Affiliations:** 1 Laboratory for Structural Neuropathology, Brain Science Institute, RIKEN, 2-1 Hirosawa, Wako-shi, Saitama 351-0198, Japan; 2 Laboratory for Developmental Neurobiology, Brain Science Institute, RIKEN, 2-1 Hirosawa, Wako-shi, Saitama 351-0198, Japan; 3 Calcium Oscillation Project, ICORP-SORST, Japan Science and Technology Agency (JST), 4-1-8 Honcho, Kawaguchi, Saitama 332-0012, Japan; 4 Present address: Neuro-Oncology Branch, National Cancer Institute, National Institute of Neurological Disorders and Stroke, National Institutes of Health, 37 Convent Drive, Bethesda, MD, 20892, USA; 5 Present address: Institute of Biochemistry, Nutrition and Health Protection, Department of Biochemistry and Microbiology, Faculty of Chemical and Food Technology, Slovak University of Technology, Radlinskeho 9, 812 37, Bratislava, Slovakia

**Keywords:** Vimentin, IP3R1, IRBIT, Rho-kinase, Huntingtin, Aggregation

## Abstract

**Background:**

Huntington's Disease (HD) is a fatal hereditary neurodegenerative disease caused by the accumulation of mutant huntingtin protein (Htt) containing an expanded polyglutamine (polyQ) tract. Activation of the channel responsible for the inositol-induced Ca^2+^ release from ensoplasmic reticulum (ER), was found to contribute substantially to neurodegeneration in HD. Importantly, chemical and genetic inhibition of inositol 1,4,5-trisphosphate (IP3) receptor type 1 (IP3R1) has been shown to reduce mutant Htt aggregation.

**Results:**

In this study, we propose a novel regulatory mechanism of IP3R1 activity by type III intermediate filament vimentin which sequesters the negative regulator of IP3R1, IRBIT, into perinuclear inclusions, and reduces its interaction with IP3R1 resulting in promotion of mutant Htt aggregation. Proteasome inhibitor MG132, which causes polyQ proteins accumulation and aggregation, enhanced the sequestration of IRBIT. Furthermore we found that IRBIT sequestration can be prevented by a rho kinase inhibitor, Y-27632.

**Conclusions:**

Our results suggest that vimentin represents a novel and additional target for the therapy of polyQ diseases.

## Background

Huntington's disease (HD) is an autosomal-dominant neurodegenerative disorder caused by CAG repeat expansion coding for a polyglutamine (polyQ) sequence in the N-terminal region of the huntingtin protein (Htt). The expansion of more than 36 repeats causes misfolding of the gene product huntingtin resulting in a toxic gain-of-function [1]. Clinically, HD is characterized by chronic and progressive involuntary choreiform movements, mood disorders, cognitive impairment, and behavioral changes [2,3]. A prominent feature of this disease is progressive neurodegeneration, with neuronal intranuclear and cytoplasmic accumulation of aggregated polyQ protein [4,5]. HD pathomechanism involves a broad scale of events including dysregulation of transcription and gene expression, impairment of axonal transport and synaptic transmission and impairment of the ubiquitin proteasome system (UPS) [6,7]. Mitochondrial dysfunction leading to induction of mitochondrial apoptotic pathway has also been described in HD with Ca^2+^ mishandling and suppression of energy metabolism [8,9]. Despite an enormous effort in elucidating the pathogenesis of this disorder, effective therapies for HD have not yet been found.

Vimentin is a 57 kDa type III intermediate filament (IF) found in cells of mesenchymal origin [10,11]. While widely expressed in embryos, vimentin is replaced by other major classes of IFs in cells during terminal differentiation [12,13]. In the adult brain, vimentin expression is mostly restricted to some subpopulations of glial and vascular endothelial cells under physiological conditions [12-14]. Importantly, it has been found that vimentin expression is re-activated in mature neurons affected by Alzheimer’s disease or traumatic injury [15,16].

Degradation of misfolded proteins has been shown partly mediated by UPS. The components of UPS including the 26S proteasome and ubiquitin as well as heat shock proteins are concentrated at the centrosome [17]. When UPS is overloaded by misfolded proteins and/or it is chemically inhibited, the centromeric accumulation of these proteins increases forming aggresomes which may represent a general cellular response to dysfunctional or damaged polyubiquitinated proteins accumulation [18,19]. Another evidence of the association of aggresome formation with the accumulation and degradation of misfolded proteins has come from studies, where pathogenic polyQ proteins Htt and atrophin-1 formed inclusions at centrosomes which were surrounded by vimentin [20,21]. Vimentin is recruited to the aggresomes during UPS dysfunction and forms a cage-like structure surrounding the pericentriolar focus of aggregated protein [19].

The role of aggresomes and especially the vimentin cage in polyQ diseases progression is not clear. We hypothesized that vimentin may play a major role in polyQ proteins accumulation and aggregation and that vimentin cage may immobilize or trap not only the UPS components and chaperones, but also other important proteins at the centrosomic inclusions and thus preventing their functions elsewhere in the cell. We found that one of such proteins, IP3R1-interacting protein released with IP3 (IRBIT) is also sequestered by vimentin. IRBIT has numerous regulatory functions among which the IP3R1 activity regulation is most intriguing [22,23]. IRBIT binds to IP3-binding core domain of IP3R1 acting as a competitor to IP3 [23,24]. Absence of IRBIT sensitizes IP3R1 to IP3, which leads to an increase in Ca^2+^ release from endoplasmic reticulum [23].

IP3R1 was found to be involved in polyQ diseases pathomechanism [25,26]. In planar lipid bilayer reconstitution experiments and in primary cultures of rat striatal medium spiny neurons, IP3R1 was sensitized to IP3 by mutant forms of Htt, while normal Htt had no effect. This finding confirmed that the activation of IP3R1 by expanded polyQ Htt is a contributing factor of Ca^2+^ signaling alteration and neuronal degeneration in HD [25]. Knock-down of IP3R1 or direct chemical inhibition of the IP3R1 activity also reduced polyQ proteins accumulation and aggregation [27] and cell death [28].

Here we introduce a novel pathway of IP3R1 activity regulation, where vimentin is able to sequester IRBIT from interaction with IP3R1. Moreover, IRBIT sequestration was enhanced by the phosphomimetic S71E/S38E vimentin mutant (E2; Ser71 and Ser38 replaced with Glu). Phosphorylation of Ser71 and Ser38 is mediated by rho-associated kinases (ROCKs) [29,30]. ROCKs are Ser/Thr protein kinases, which were found to be downstream targets of the small GTPase RhoA [31,32]. In the mammalian system, ROCKs consist of two isoforms, ROCK1 and ROCK2 [31]. They are important regulators of cell growth, migration, and apoptosis via control of actin cytoskeletal assembly [33]. Blocking the RhoA/ROCK pathway has been shown to inhibit the polyQ protein aggregation and decrease its toxicity in cellular and Drosophila models of HD [34]. ROCK1 and protein kinase C-related protein kinase-2 (PRK-2) have been identified to be the mediators of aggregation reduction by the well-known ROCK inhibitor Y-27632 [35]. Moreover, a downstream effector of ROCK1, actin-binding factor profilin, was reported to inhibit the mutant Htt aggregation by direct interaction via its polyproline-binding domain [36]. Previously, we have reported that Y-27632 treatment also reduced aggregation of several other polyQ proteins without polyproline tracts, thus possibly affecting additional targets [37,38]. Here we show that vimentin may represent one of the mediators of ROCK inhibition-dependent reduction of pathogenic polyQ proteins aggregation via modulation of IP3R1 activity by IRBIT.

## Results and discussion

### Effect of vimentin levels and phosphorylation on polyQ aggregation

To investigate the role of vimentin in polyQ Htt processing, we considered several clues. Firstly, UPS impairment is thought to contribute to the severity of HD [6,7]. Vimentin creates cages around aggresomes, which are formed in response to accumulation of misfolded proteins and UPS dysfunction [19]. Secondly, vimentin is phosphorylated by several kinases, including ROCKs [29,30]. Thirdly, ROCK1 is activated by dopamine through dopamine D2 receptor (D2R)-specific pathway potentiating the glutamate excitotoxicity in HD [39,40] and the genetic and chemical inhibition of Rho/ROCK signaling pathway reversed dopamine/D2R-mediated cellular pathology [40]. Importantly, ROCK inhibitor Y-27632 reduced mutant Htt levels and aggregation both *in vitro*[34,37] and *in vivo* and improved motor impairment in R6/2 HD mouse model [41].

**Figure 1:**
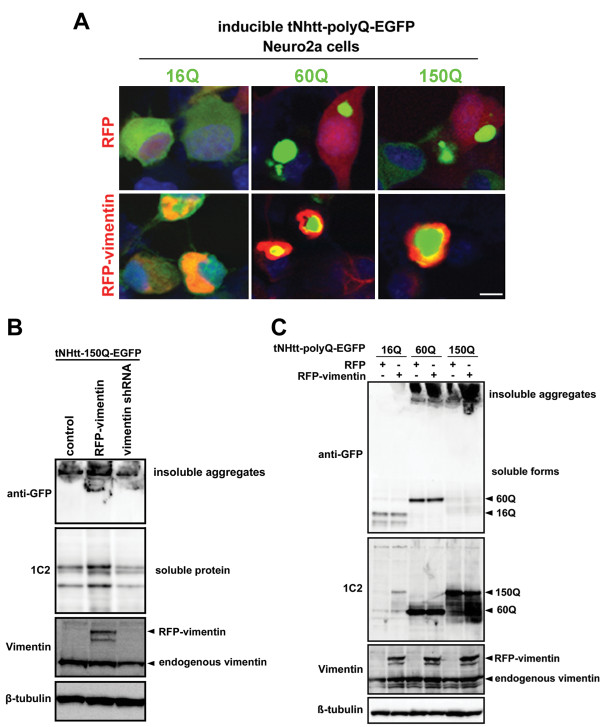
**Vimentin modifies mutant Htt aggregation. A**. Representative confocal images show distribution of normal (16Q) and pathogenic (60Q and 150Q) tNHtt (green) and RFP or RFP-vimentin (red) in inducible tNHtt-polyQ-EGFP Neuro2a cells. Note the cages formed by vimentin in 60Q and 150Q Neuro2a cells. Nuclei were stained with DAPI (blue). Scale bar, 5 μm. **B**. RFP-vimentin expression increased and vimentin knock-down reduced polyQ aggregation and levels of total mutant Htt in 150Q Neuro2a cells as compared to the control. **C**. The effect of RFP-vimentin on Htt levels is polyQ length-dependent. While tNHtt-60Q-EGFP and tNHtt-150Q-EGFP accumulated as the insoluble forms at the gel top, tNHtt-16Q-EGFP levels remained unchanged upon RFP-vimentin transfection.

We overexpressed RFP or RFP-vimentin in 16Q and 60Q and 150Q Neuro2a cells. We observed that vimentin accumulated at perinuclear regions and formed cage-like structures around tNHtt-60Q-EGFP and tNHtt-150Q-EGFP inclusions in 60Q and 150Q Neuro2a cells while RFP exerted diffuse distribution in all cell lines (Figure 1A and Additional file 1: Figure S1). This confirmed the previously reported colocalization of vimentin with pathogenic polyQ protein inclusions [17,18].

Next we asked whether vimentin could modulate mutant Htt aggregation. We found that over-expression of RFP-vimentin in 150Q Neuro2a cells dramatically increased the accumulation of insoluble Htt. Accumulation of the soluble form was also observed and could be the result of enhanced aggresomes formation leading to suppression of UPS activity under this condition. Vimentin knock-down, on the other hand reduced the mutant Htt aggregation (Figure 1B). To test whether the effect of vimentin is polyQ length-dependent, we over-expressed RFP-vimentin in 16Q, 60Q and 150Q Neuro2a cells. Vimentin appeared to act specifically on mutant Htt, as the levels of tNHtt-16Q-EGFP remained unchanged while the accumulation of insoluble pool of the pathogenic Htt forms increased (Figure 1C).

**Figure 2:**
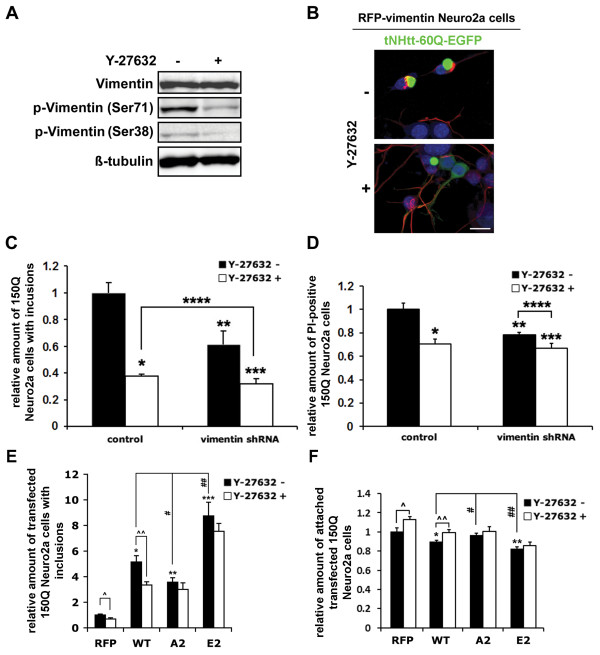
**Vimentin affects the mutant Htt inclusion formation in 150Q Neuro2a cells and mediates the effect of Y-27632. A**. Immunoblot demonstrating inhibition of vimentin phosphorylation at Ser71 and Ser38 by ROCK inhibitor Y-27632 (20 μM) in Neuro2a cells. **B**. tNHtt-60Q-EGFP (green) was transfected to Neuro2a cells stably expressing RFP-vimentin (red). Treatment of these cells with 20 μM Y-27632 resulted in filament-like distribution of vimentin and disruption of vimentin cages observed around tNHtt-60Q-EGFP inclusions in the untreated cells. Nuclei were stained with DAPI (blue). Scale bar, 15 μm. **C**. The effect of Y-286432 on polyQ inclusion formation depends on vimentin level (high vimentin levels enhance inclusion formation). 150Q Neuro2a cells were transfected with vimentin shRNA and 48 hrs later, the cells were induced and treated with 20 μM Y-27632. After 24 hrs, cells were fixed and the inclusion formation was quantified by ArrayScan. *p = 0.00003, ** *p* = 0.0012, ***p = 0.00003, ****p = 0.0018. **D**. The effect of vimentin knock-down and Y-27632 on polyQ cytotoxicity. Cells were incubated with PI and the PI-positivity was quantified by ArrayScan. *p = 0.0022, ** *p* = 0.002, ***p = 0.001, ****p = 0.017. **E**. The effect of Y-286432 on polyQ inclusion formation is dependent on vimentin phosphorylation (vimentin phosphorylation enhances inclusion formation). 150Q Neuro2a cells were transfected with RFP, WT and phospho-mutant (A2 and E2) forms of RFP-vimentin. Cells were induced and treated with 20 μM Y-27632 for 24 hrs, fixed and analyzed by ArrayScan. *p = 0.00016, **p = 0.0003, ***p = 0.0004, ^ˆ^*p* = 0.0021, ^ˆˆ^*p* = 0.018, ^#^*p* = 0.04, ^##^*p* = 0.022. **F**. The effect of vimentin over-expression and Y-27632 on polyQ-induced cell death. Dead cells were detached and removed during samples preparation for analysis. Cells that remained attached were counted by ArrayScan. *p = 0.049, **p = 0.008, ^ˆ^*p* = 0.044, ^ˆˆ^*p* = 0.045, ^#^*p* = 0.032, ^##^*p* = 0.034. Bars in **C-F** represent relative mean values ± s.d. from three independent experiments, with levels under control conditions normalized to a value of 1.

Vimentin has been shown phosphorylated by ROCK at Ser71 and Ser38 amino residues [29,30] and we confirmed this fact, as treatment of Neuro2a cells with the ROCK inhibitor Y-27632 reduced the phosphorylation at these sites (Figure 2A). We transfected stable RFP-vimentin Neuro2a cells with tNHtt-60Q-EGFP and treated them with Y-27632. Interestingly, we detected a modified subcellular distribution of stably expressed RFP-vimentin in Neuro2a cells treated with Y-27632 (Figure 2B). In the untreated cells, RFP-vimentin formed cage-like structures around tNHtt-60Q-EGFP inclusions while the Y-27632 treatment changed the localization of RFP-vimentin to neurites (Figure 2B). This observation suggested that vimentin phosphorylation by ROCK might influence polyQ aggregation.

To quantify the effects of vimentin levels on mutant Htt aggregation, we transfected the 150Q Neuro2a cells with vimentin shRNA and counted the inclusions on a cell-to-cell basis by ArrayScan. Vimentin knock-down reduced the number of the cells with inclusions by 39% (Figure 2C). Treatment of the 150Q Neuro2a cells with 20 μM Y-27632 reduced the polyQ aggregation by 62%, similarly to the previously reported effect in these cells [37]. Vimentin knock-down significantly decreased the effect of Y-27632 to 40% (22% difference as compared to the 62% aggregation reduction in the non-transfected cells) (Figure 2C), suggesting that the effect of Y-27632 is partly mediated through the inhibition of the phosphorylation of vimentin. Importantly, vimentin knock-down also significantly decreased the number of propidium iodide (PI)-positive 150Q Neuro2a cells indicating reduction of the polyQ toxicity (Figure 2D). We next analyzed the anti-aggregation effect of WT and phospho-mutants of vimentin in 150Q Neuro2a cells. Ser71 and Ser38 were substituted with phosphomimetic Glu (E2 mutant) or non-phosphorylated Ala (A2 mutant) amino acid residues. Over-expression of any of the RFP-vimentin form increased inclusion formation in 150Q Neuro2a cells. The E2 and A2 mutants had significantly stronger and weaker effect, respectively, as compared to the WT vimentin. Importantly, the effect of Y-27632 was abolished in cells expressing vimentin mutants (Figure 2E). WT and E2 mutants significantly increased the number of dead cells removed from the wells during the preparation of the samples for ArrayScan analysis while A2 vimentin did not have significant effect as compared to the control cells transfected with RFP (Figure 2F). These results confirmed that the effect of ROCK inhibitor Y-27632 on the mutant Htt aggregation and cytotoxicity is mediated by the phosphorylation status of vimentin and partly depends on the levels of this protein.

### Vimentin sequesters IRBIT and decreases its interaction with IP3R1

Next, we aimed to identify the mechanism, by which vimentin levels and phosphorylation modifies accumulation and aggregation of pathogenic Htt. Our hypothesis on vimentin affecting polyQ aggregation in cooperation with IP3R1 was based on several studies. Firstly, the phosphorylation dynamics plays an important role in vimentin network reorganization and it changes vimentin affinity to its interacting partners, mostly regulatory proteins, and their spatial distribution [42]. Secondly, IP3R1 is directly involved in mutant Htt inclusion formation [27]. Thirdly, there has been reported a crosstalk between IP3R1 activity and intermediate filaments [43]. It has also been suggested that IP3Rs may be involved in the mechanism underlying the potentiating action of the Y-27632 in neurite outgrowth [44], which includes modifications of vimentin dynamics [45].

**Figure 3:**
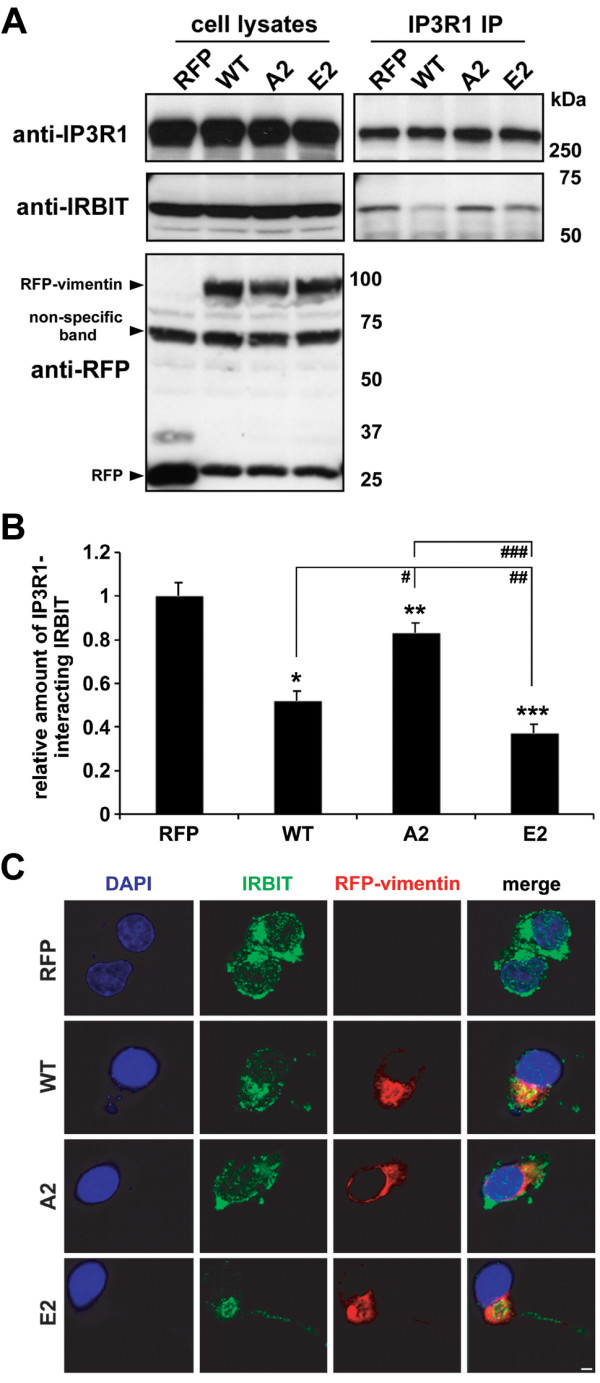
**Vimentin influences the IRBIT-IP3R1 interaction by sequestering IRBIT in perinuclear inclusions. A**. HeLa cells were transfected with RFP or tested forms of RFP-vimentin. 24 hrs later, cells were lysed and immunopreciptitation was performed using 10A6 anti-IP3R1 antibody. For immunoblotting, KM1112 anti-IP3R1, anti-IRBIT and anti-RFP antibodies were used. **B**. Quantification of IRBIT-IP3R1 interaction. The densities of immunoprecipitated IRBIT were normalized to the densities of corresponding immunoprecipitated IP3R1. *p = 0.0002, **p = 0.009, ***p = 0.0001, ^#^*p* = 0.0017, ^##^*p* = 0.029, ^###^*p* = 0.0009. Bars represent relative mean values ± s.d. from three independent experiments, with levels of IRBIT-IP3R1 interaction under control conditions normalized to a value of 1. **C**. Representative confocal images show distribution of immobile fraction of IRBIT in presence of RFP or tested RFP-vimentin forms. Neuro2a cells were transfected with RFP or RFP-vimentins, 24 hrs later permeabilized with saponin and immunostained for endogenous IRBIT (green). Fluorescence of RFP (red) and DAPI (blue) are also shown. Note that RFP was washed out from cells during saponin permeabilization. Scale bar, 5 μm.

As inhibiting IP3R1 activity reduced polyQ Htt accumulation and aggregation [27], it was feasible to assume that stimulation of IP3R1 can contribute to polyQ aggregation. While exploring this hypothesis, we focused on one of the IP3R regulatory proteins, IRBIT [46]. IRBIT binds to IP3R1 and prevents its activation by IP3 [23]. HeLa cells were transfected with RFP or tested forms of RFP-vimentin and 24 hrs later, immunoprecipitation using anti-IP3R1 antibody was performed. We found that over-expression of RFP-vimentin suppressed the IRBIT-IP3R1 interaction by 47% (Figure 3A, B). As the phosphorylation status of vimentin turned out important for the extent of the polyQ aggregation modification (Figure 2), we also wondered how the vimentin phospho-mutants A2 and E2 would influence the IRBIT-IP3R1 interaction. As expected, the A2 mutant reduced this interaction only by 18% while the E2 form of vimentin suppressed the IRBIT binding to IP3R1 by 63% as compared to the control (Figure 3A, B). These findings suggested that both the levels and phosphorylation status of vimentin are determining factors in suppressing the IRBIT-IP3R1 interaction and therefore influencing the activity of IP3R1.

To support our observations, we investigated the localization of the membrane-bound fraction of IRBIT in the presence of vimentin. We transfected Neuro2a cells with RFP or with tested RFP-vimentin forms. The cells were then permeabilized with saponin to remove the soluble cytosolic proteins, and subjected to confocal microscopy with immunostained IRBIT. While RFP, as a soluble protein not interacting with cytoskeleton or membranes, was not detected in the samples, RFP-vimentin was present and displayed different localization patterns depending on the amino acids at positions 71 and 38. WT and particularly E2 vimentin formed perinuclear cage-like structures, while the A2 mutant was dispersed with mostly filamentous-like distribution (Figure 3C). Importantly, IRBIT appeared trapped inside the structures formed by WT and E2 RFP-vimentins with almost exclusive localization of IRBIT within these inclusions in the E2-transfected cells. The A2 mutant, on the other hand, did not affect the IRBIT distribution so markedly as compared to the control RFP-transfected cells (Figure 3C). These observations are in agreement with the data obtained by IRBIT-IP3R1 co-immunoprecipitation (Figure 3A, B).

### Modification of IRBIT sequestration by ROCK and UPS inhibition

**Figure 4:**
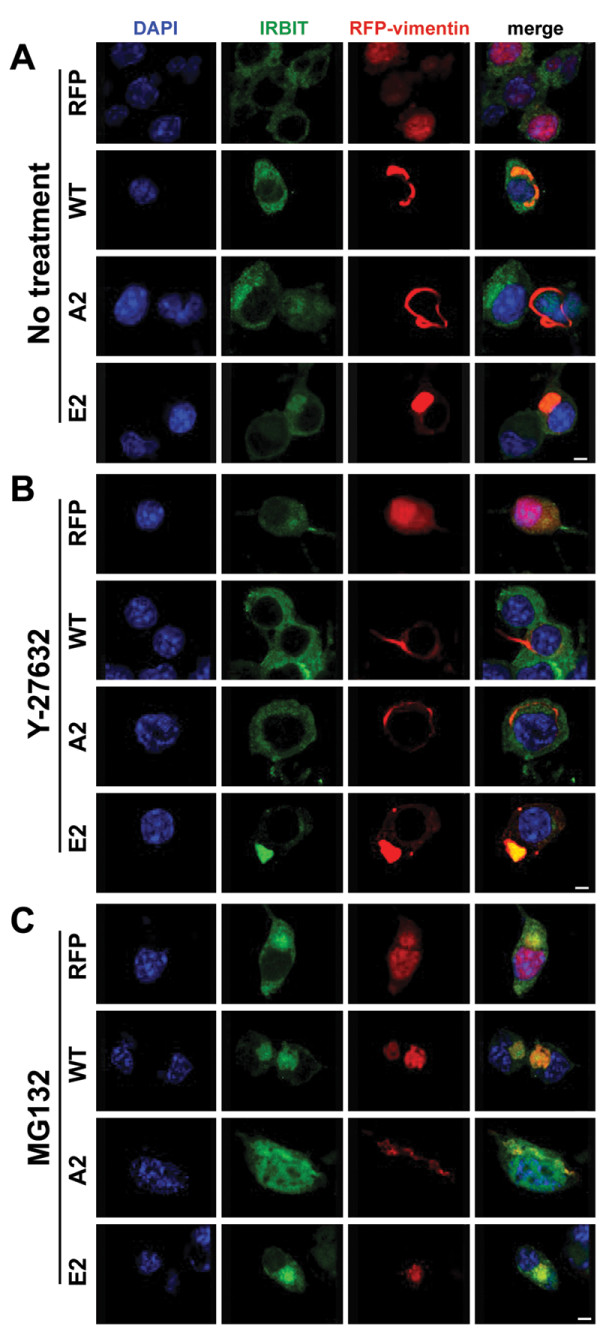
**Vimentin phosphorylation affects IRBIT subcellular distribution. Effect of ROCK and UPS inhibitors.** Representative confocal images show subcellular distribution of IRBIT in presence of RFP or tested RFP-vimentin forms. Neuro2a cells were transfected and treated as indicated for 24 hrs. Cells were fixed, permeabilized and immunostained for enogenous IRBIT (green). **A**. Control untreated cells (DMSO, 1/1000). **B**. Cells treated with 20 μM Y-27632. **C**. Cells treated with 5 μM MG132. Fluorescence of RFP (red) and DAPI (blue) are also shown. Scale bar, 5 μm.

The next question was whether the distribution of tested RFP-vimentins and IRBIT could be modified upon ROCK or UPS inhibition by Y-27632 or MG132 treatment, respectively. In the untreated cells, E2 vimentin accumulated in perinuclear inclusions and colocalized with IRBIT. Diffuse cytoplasmic staining of IRBIT was markedly reduced as compared to control cells indicating that IRBIT was recruited by E2 mutant to the aggresome-like inclusions. The A2 mutant exerted filamentous-like distribution with most of IRBIT remaining diffuse. The distribution of WT vimentin appeared as an intermediate pattern between the mutants (Figure 4A). When cells were treated with Y-27632, the WT form gained a filamentous distribution (similar to A2 mutant at this and at the control condition) and lost the colocalization with IRBIT seen in the non-treated cells (Figure 4B). The unphosphorylated A2 and phosphomimetic E2 mutants were resistant to Y-27632 treatment (target serine amino acids mutated) and retained their distributions with A2 being filamentous-like, and E2 forming perinuclear aggresome-like inclusions and trapping IRBIT. These results suggest that the subcellular distribution of IRBIT regulated by vimentin is under the control of ROCK through phosphorylation of Ser71 and Ser38.

UPS inhibition has been shown to induce the formation of aggresomes [20]. Upon treatment with MG132, we observed IRBIT accumulation in aggresome-like structures even in control cells transfected with RFP. UPS inhibition in cells expressing WT RFP-vimentin caused its complete relocation into perinuclear inclusions and IRBIT accumulation in this location was markedly enhanced as compared to control cells, resembling the effect of E2 RFP-vimentin (Figure 4C). In the E2-transfected cells, this distribution of vimentin and IRBIT was observed under all conditions (Figure 4A-C). Unexpectedly, the A2 mutant appeared to be resistant to MG132 treatment, retained its filamentous structure and prevented the accumulation of IRBIT in the aggresome-like inclusions (Figure 4C). This observation suggested a novel role for vimentin as a component actively regulating aggresome formation or at least sequestering and immobilizing certain proteins within this structure. We hypothesize that when the vimentin cage is not fully formed, some of the proteins can escape from aggresomes and at least partially fulfill their function at the physiological subcellular locations.

Overall, our results suggest that IRBIT can be sequestered by vimentin to perinuclear aggresome-like structures. The extent of sequestration appears to depend not only on the levels but also on the phosphorylation status of vimentin. All the above-discussed observations on vimentin-IRBIT connection were obtained in the absence of mutant Htt to avoid possible influence of this pathogenic protein, as mutant Htt sensitizes IP3R1 to IP3 via direct binding to the C-terminal part of IP3R1 [25,39] and augmenting aggresome formation [19].

### Effect of vimentin on mutant Htt aggregation is mediated by IRBIT

**Figure 5:**
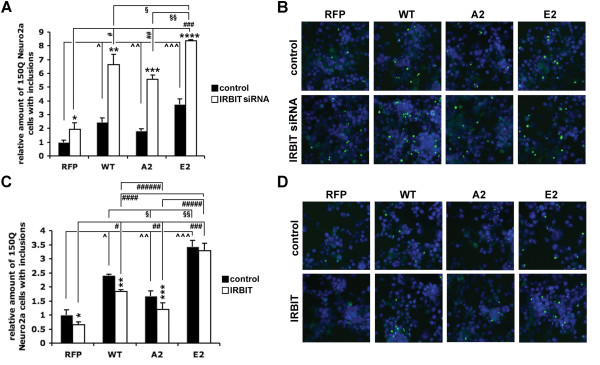
**IRBIT modifies inclusion formation in 150Q Neuro2a cells. A**. 48 hrs after transfection of control or IRBIT siRNA and RFP or tested RFP-vimentin forms, cells were induced for 24 hrs, fixed, and analyzed by ArrayScan. *p = 0.028, **p = 0.0008, ***p = 0.00005, ****p = 0.00004, ^ˆ^*p* = 0.0018, ^ˆˆ^*p* = 0.0021, ^ˆˆˆ^*p* = 0.00034, ^#^*p* = 0.0007, ^##^*p* = 0.0004, ^###^*p* = 0.00002, ^#167;^*p* = 0.016, ^#167;#167;^*p* = 0.0001. **B**. Images generated and analyzed by ArrayScan representing each experimental condition in A. Green, tNHtt-150Q-EGFP; blue, Hoechst 33258. Magnification, 20x. **C**. Cells were co-transfected with mock or flag-IRBIT and RFP or tested RFP-vimentin forms and induced for 24 hrs before analyzed by ArrayScan. *p = 0.042, **p = 0.0002, ***p = 0.049, ^ˆ^*p* = 0.0002, ^ˆˆ^*p* = 0.0099, ^ˆˆˆ^*p* = 0.00013, ^#^*p* = 0.00005, ^##^*p* = 0.015, ^###^*p* = 0.00009, ^####^*p* = 0.0008, ^#####^*p* = 0.0005, ^######^*p* = 0.0092, ^#167;^*p* = 0.0026, ^#167;#167;^*p* = 0.0016. **D**. Images generated and analyzed by ArrayScan representing each experimental condition in C. Green, tNHtt-150Q-EGFP; blue, Hoechst 33258. Magnification, 20x. Bars in **A** and **C** represent relative mean values ± s.d. from four independent experiments, with levels of inclusion formation under control conditions normalized to a value of 1.

To test the relevance of the vimentin-IRBIT pathway to HD, we examined whether IRBIT could be a mediator of the modifying effect of vimentin on mutant Htt aggregation. We over-expressed WT, A2 or E2 RFP-vimentin in 150Q Neuro2a cells with or without knocking-down IRBIT, induced the tNHtt-150Q-EGFP expression and analyzed the inclusion formation by ArrayScan. Knock-down of IRBIT increased the inclusion formation almost twice (1.95-fold) in the RFP-expressing cells. The effect of IRBIT knock-down was enhanced by all forms of vimentin with E2 mutant having the strongest effect followed by WT and the A2 mutant with 8.37-, 6.65- and 5.57-fold increase of inclusion formation, respectively (Figure 5A). Over-expression of IRBIT, in contrary, reduced the inclusion formation in 150Q Neuro2a cells by 35%. The effect of high IRBIT levels was relatively reduced in the presence of WT and A2 vimentin, with 23% and 28% difference, respectively, between the single and double transfections. E2 vimentin, on the other hand, abolished the effect of IRBIT with no difference between the E2 and E2 + IRBIT conditions (Figure 5C). Examples of compound images generated by ArrayScan representing each experimental condition are shown in Figure 5B and D.

These data suggest that over-expressed WT and particularly E2 vimentin sequester IRBIT and may impair its function, while the A2 mutant has a relatively mild inhibitory effect. This is in accordance with the results in Figures 3 and 4 showing phosphorylated vimentin trapping IRBIT in perinuclear structures more efficiently than the phospho-resistant, A2 form.

## Conclusions

In the present study, we introduce vimentin as a modifier of mutant Htt aggregation. Vimentin over-expression increased and the knock-down reduced the mutant Htt aggregation in Neuro2a cells. ROCK inhibitor Y-27632 inhibited vimentin phosphorylation at Ser71 and Ser38 and reduced the promoting effect of vimentin on mutant Htt aggregation. We found that interaction of IRBIT with IP3R1 is affected by vimentin and that the extent of this effect is dependent on the amino acids at positions 71 and 38. Accordingly, vimentin sequestered IRBIT in cage-like structures resembling aggresomes with the phosphomimetic E2 vimentin mutant traping IRBIT almost exclusively in perinuclear inclusions. The unphosphorylated A2 mutant expression, on the other hand, did not result in cage formation and IRBIT sequestration even when UPS was inhibited. We showed the relevance of vimentin-IRBIT axis in polyQ aggregation regulation in 150Q Neuro2a cells, where reduced levels of IRBIT enhanced, and increased levels of IRBIT decreased mutant Htt inclusion formation. These effects were modified by vimentin levels and mutations at Ser71 and Ser38. Although it has been speculated that aggresomes fulfill a protective role in polyQ diseases pathomechanism [47], based on our study we hypothesize that this function might depend on the dynamics of aggresome formation. If normal cellular proteins are sequestered too fast to the aggresomes without a sufficient time period for the cell to replace them or adapt to this state, it may contribute to cell death.

**Figure 6:**
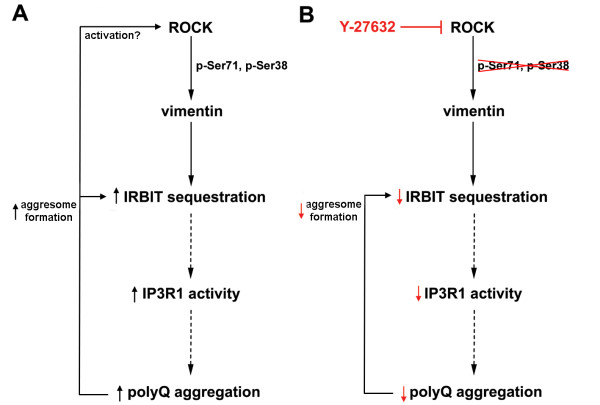
**Schematic representation of the proposed mechanism of the vimentin effect on mutant Htt aggregation. A**. In HD, mutant Htt and dopamine stimulation may activate ROCK, which in turn phosphorylates vimentin at Ser71 and Ser38 leading to IRBIT sequestration, reduced interaction of IRBIT with IP3R1, and activation of IP3R1. Mutant Htt expression and aggregation may increase aggresome formation enhancing IRBIT sequestration. **B**. Blocking vimentin phosphorylation by ROCK inhibitors may lead to reduced IRBIT sequestration by vimentin and consequently to decreased accumulation and aggregation of the pathogenic polyQ protein.

We would like to propose the following mechanistic model for the modifying effect of vimentin on polyQ protein accumulation and inclusion formation: vimentin and preferentially its phosphorylated form (at Ser71 and Ser38) promote polyQ aggregation by sequestering IRBIT in perinuclear inclusions and preventing its interaction with IP3R1 (Figure 6A). Increased activity of IP3R1 in polyQ diseases models has been previously reported to contribute to cytotoxicity of the pathogenic misfolded proteins [25,26,28] and knock-down or chemical inhibition of IP3R1 reduced mutant Htt aggregation [27]. The therapeutic potential of ROCK inhibitors may thus be partly mediated by decreased vimentin phosphorylation leading to reduction of IP3R1 activity (Figure 6B). Importantly, activation of vimentin expression was shown in mature neurons affected by neurodegenerative or traumatic insults [15,16]. Reduction of vimentin levels and/or phosphorylation appears as a promising therapeutic strategy for HD and other polyQ diseases.

## Methods

### Materials

The ROCK inhibitor Y-27632 was from Sigma and MG132 (Z-Leu-Leu-Leu-aldehyde) from Wako Chemicals. Fluorescent nucleic acid stain Hoechst 33258 was from Molecular Probes. Mouse monoclonal antibody recognizing expanded polyQ tract, 1C2, and rat monoclonal anti-β-tubulin were obtained from Chemicon. Mouse monoclonal anti-vimentin antibody antibody was from Sigma. Rat monoclonal anti-phospho-vimentin (Ser71) and (Ser38) antibodies, and mouse monoclonal anti-GFP and anti-RFP antibodies were purchased from MBL. Mouse monoclonal anti-IP3R1 antibody KM1112, rat anti-IP3R1 antibody 10A6 and rabbit anti-IRBIT were generated as reported previously [22,48,49].

### Plasmids

Plasmids encoding the truncated N-terminal of human huntingtin (tNHtt) with 16, 60, and 150 glutamine repeats were introduced in pEGFP-N1 vector as previously described [50]. To prepare pcDNA3.1-tNHtt-polyQ-EGFP with 60Q and 150Q for transient transfection, tNHtt-polyQ-EGFP fragment was cut from pIND tNHtt-polyQ-EGFP [51] with HindIII-XbaI digestion, and the resulting fragment was inserted into pcDNA3.1-v5/His plasmid. The monomeric red fluorescence protein (RFP) plasmid preparation has previously been described [52]. Construction of N-terminally Flag-tagged IRBIT was described previously [23].

Mouse vimentin was amplified from mouse cDNA library using 5’-TCCCGAATTCAAGCTTCCACCATGTCTACCAGGTCTGTGTCC-3’ as forward and 5’-AAACACCGGATCCGGTTCAAGGTCATCGTGATGCTG-3’ as reverse primer. The amplified vimentin cDNA fragment was inserted into HindIII/BamHI site of the pmRFP-C1 plasmid and named RFP-vimentin.

The mutations in RFP-vimentin were introduced using QuikChange® Site-Directed Mutagenesis Kit (Stratagene). To generate phospho-mimetic (E2) and unphosphorylated (A2) mutants, following primers were used to exchange Ser71 and Ser38 to Glu or Ala: Ser71Glu: forward: 5`-GTGCGCCTGCGGGAAAGCGTGCCGGGCTG-3` and reverse, 5`-CAGCCCGGCACGCTTTCCCGCAGGCGCAC-3` Ser38Glu: forward, 5`- CACGTCCACACGCACCTACGAACTGGGCAGCGCAC-3` and reverse, 5`- GTGCGCTGCCCAGTTCGTAGGTGCGTGTGGACGTG-3; Ser71Ala: forward, 5`-GTGCGCCTGCG GGCTAGCGTGCCGGGCTG-3` and reverse, 5`-CAGCCCGGCACGCTAGCCCGCAGGCGCAC-3`. Ser38Ala: forward, 5`- CACGTCCACACGCACCTACGCTCTGGGCAGCGCAC-3` and reverse, 5`- GTGCGCTGCCCAGAGCGTAGGTGCGTGTGGACGTG-3`.

### Cell culture, transient transfection and treatments

Mouse neuroblastoma (Neuro2a) and human cervical carcinoma cells (HeLa) cells were maintained in Dulbecco's modified Eagle's medium (Sigma) supplemented with 10% heat-inactivated fetal bovine serum (Sigma), 100 U/ml penicillin and 100 μg/ml streptomycin (Invitrogen) at 37°C in an humidified atmosphere containing 5% CO_2_. Establishment of stable Neuro2a cell lines with the ecdysone-inducible mammalian expression system (Invitrogen), that express tNHtt-EGFP-16Q (16Q Neuro2a cells), tNHtt-60Q-EGFP (60Q Neuro2a cells) and tNHtt-EGFP-150Q (150Q Neuro2a cells) has been described earlier [50,51]. Neuro2a cells were differentiated with 5 mM dbcAMP (*N*^6^,2'-*O*-dibutyryladenosine-3',5'-cyclic monophosphate sodium salt) and induced to express tNHtt-polyQ-EGFP with 2 μM ponasterone A (Invitrogen).

RFP-vimentin was transfected into Neuro2a/FRT cells [53]. The stably transfected cells resistant to treatment with 400 μg/ml G418 (Calbiochem), were sub-cloned twice.

All transient transfections were performed when the cells reached 70-80% confluence with Lipofectamine 2000 (Invitrogen) or Trans-IT (Mirus) according to the manufacturer’s instruction.

### RNA interference

The non-silencing control, vimentin and IP3R1 shRNAs were obtained from Open Biosystems. Plasmids were transfected into cells using Lipofectamine 2000. Neuro2a cells were induced 48 hrs later. Stealth siRNA specific for IRBIT and scrambled control were obtained from Invitrogen. 20 μM siRNA stock solutions were used for transfection to Neuro2a cells by Lipofectamine 2000 and after 48 hrs, cells were transfected again with RFP or RFP-vimentin. Cells were used for experiments 24 hrs later.

### ArrayScan quantification

For the quantification of the inclusions, cells were grown in 24-well plates, fixed in 4% paraformaldehyde, washed and incubated with Hoechst 33258 at 1/1000 dilution in PBS. Cells were analyzed by ArrayScan®V^TI^ High Content Screening (HCS) Reader (Cellomics) using Target Activation BioApplication (TABA) as described earlier [37]. TABA analyzes images acquired by a HCS Reader and provides measurements of the intracellular fluorescence intensity and localization on a cell-by-cell basis. In each well, at least 10,000 cells were counted and quantified for the presence of the inclusions. Scanning was performed with triplicate or quadruplicate in each experimental condition.

### Cell death assay

For quantification of cell viability, 5 μg/ml each of Hoechst 33342 and PI were added to differentiated and induced Neuro2a cells. After 10 min at 37°C, the PI-positive cells were quantified with ArrayScan.

### HeLa cells lysis and immunoprecipitation experiments

Twenty four hours after transfection, HeLa cells were lysed in buffer containing 50 mM Hepes (pH 7.5), 150 mM NaCl, 2 mM EDTA, Complete protease inhibitor cocktail (Roche) and 0.5% NP40 (Sigma) for 30 min on ice and briefly sonicated. Cell lysates were centrifuged at 10,000 g for 30 min at 4°C. Supernatants were rotated for 2 hrs at 4°C with IP3R1 antibody. Immuno-bound complexes were isolated by incubation with 20 μl of protein G-Sepharose 4B beads (50% slurry) (Amersham) for 2 hrs at 4°C. Precipitated proteins were eluted with SDS-PAGE sample buffer and analyzed by western blotting with appropriate antibodies.

### Western blotting

Cells were washed twice with ice-cold PBS, scraped, and resuspended in lysis buffer containing 0.5% Triton X-100 in PBS (pH 7.4), 0.5 mM phenylmethylsulfonyl fluoride and Complete protease inhibitor cocktail. After incubating on ice for 30 min, lysates were briefly sonicated. Protein concentrations were determined according to the method of Bradford using Bio-Rad protein assay reagent (Bio-Rad) and the Western blot procedure was performed as described previously [37]. Images were quantified using Multi Gauge software (Fujifilm).

### Confocal microscopy

Neuro2a cells were grown, transfected and treated in 4-well chamber slides. Cells were processed according to two protocols. Firstly, permeabilization and fixation protocol was used to wash out cytosolic proteins not bound to membranes or cytoskeleton. Cells were washed with PBS followed incubation in ice-cold pre-extraction buffer containing 80 mM PIPES (pH 7.2), 1 mM MgCl_2_, 1 mM EGTA, 4% PEG 6000 and 0.1% saponin on ice for 10 min. Samples were rinsed with PBS and fixed with 4% formaldehyde in PBS for 15 min at room temperature. Secondly, standard procedure was used with cell fixation in 4% paraformaldehyde/PBS and permeabilization with 0.1% Triton X-100/PBS. Samples were incubated with anti-IRBIT antibody for 1 hr at room temperature, washed, incubated for 1 hr with Alexa Fluor 488 anti-rabbit secondary antibody (Invitrogen) and mounted with Vectashield mounting medium containing DAPI. Inducible tNHtt-polyQ-EGFP Neuro2a transfected with RFP or RFP-vimentin and Neuro2a cells stably expressing RFP-vimentin transfected with tNHtt-16Q-EGFP or tNHtt-60Q-EGFP were fixed using 4% paraformaldehyde/PBS, and mounted with Vectashield mounting medium containing DAPI. Images were generated using confocal microscope (Leica).

### Statistical analysis

Unpaired student’s *t*-test for comparison between two samples was used. One-way ANOVA Fisher's test followed by Tukey's HSD test or two-way ANOVA test with pair-wise contrast was performed. The data was generated with XLSTAT software. We considered the difference between comparisons to be significant when *p* < 0.05 for all statistical analyses.

## Abbreviations

HD: Huntington’s Disease; Htt: Huntingtin; polyQ: Polyglutamine; IP3: Inositol 1,4,5-trisphosphate; IP3R1: IP3 receptor type 1; IRBIT: IP3R1-interacting protein released with IP3; UPS: Ubiquitin proteasome system; IF: Intermediate filament; WT: Wild-type; D2R: Dopamine D2 receptor; ROCK: Rho-associated kinase; PRK-2: Protein kinase C-related protein kinase-2; tNHtt: Truncated N-terminal of human Htt; EGFP: Enhanced green fluorescent protein; RFP: Red fluorescence protein.

## Competing interests

The authors declare that they have no competing interests.

## Authors’ contributions

POB and RH raised the hypothesis and designed the experiments. Experimental work was performed by POB, RH, AG and MK RFP-vimentin construct and the stable RFP-Vimentin Neuro2a cell line was prepared by GM The results were analyzed by POB, RH, AG, KM and NN The manuscript was written by POB, RH and NN. All authors discussed the results and commented on the manuscript.

## Supplementary Material

Additional file 1 **Figure S1. **Confocal images show distribution of pathogenic 150Q tNHtt (green) and RFP-vimentin (red) in inducible tNHtt-150Q-EGFP Neuro2a cells. Note the cages formed by vimentin around the 150Q tNHtt aggregates. Nuclei were stained with DAPI (blue). Scale bar, 10 μm. Click here for file
