# Supplementary material for: ROCK-phosphorylated vimentin modifies mutant huntingtin aggregation via sequestration of IRBIT
Source: Mol Neurodegener. 2012 Aug 28;7:43. doi: 10.1186/1750-1326-7-43 (PMC3502191; doi:10.1186/1750-1326-7-43)
Supplement: Additional file 1 — Figure S1. Confocal images show distribution of pathogenic 150Q tNHtt (green) and RFP-vimentin (red) in inducible tNHtt-150Q-EGFP Neuro2a cells. Note the cages formed by vimentin around the 150Q tNHtt aggregates. Nuclei were stained with DAPI (blue). Scale bar, 10 μm. [file 1750-1326-7-43-S1.pdf]

Additional data

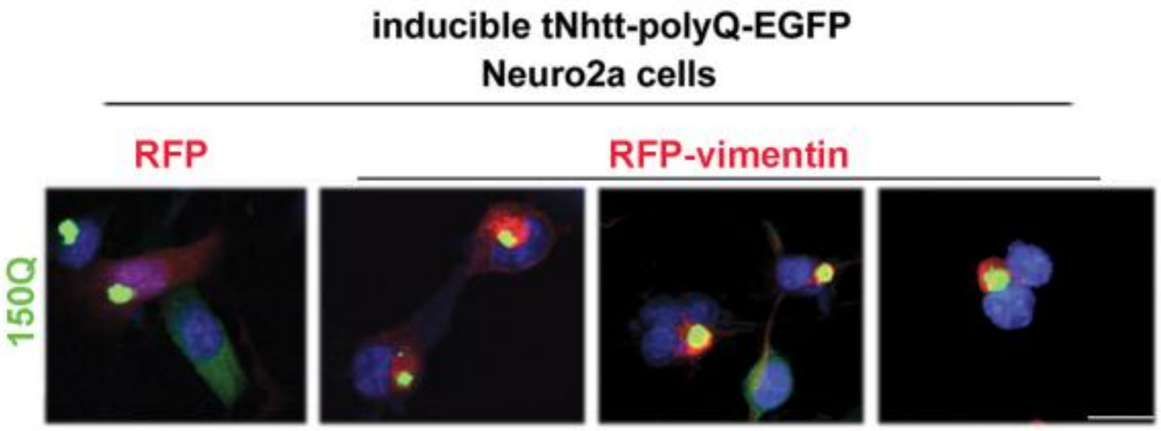

Supplementary Figure 1. Confocal images show distribution of pathogenic 150Q tNHtt (green) and RFP-vimentin (red) in inducible tNHtt-150Q-EGFP Neuro2a cells. Note the cages formed by vimentin around the 150Q tNHtt aggregates. Nuclei were stained with DAPI (blue). Scale bar, 10 μm.
